# Reinforcement learning as an innovative model-based approach: Examples from precision dosing, digital health and computational psychiatry

**DOI:** 10.3389/fphar.2022.1094281

**Published:** 2023-02-17

**Authors:** Benjamin Ribba

**Affiliations:** Roche Pharma Research and Early Development (pRED), F. Hoffmann-La Roche Ltd, Basel, Switzerland

**Keywords:** pharmacometrics, digital health, reinforcement learning, precision dosing, computational psychiatry

## Abstract

Model-based approaches are instrumental for successful drug development and use. Anchored within pharmacological principles, through mathematical modeling they contribute to the quantification of drug response variability and enables precision dosing. Reinforcement learning (RL)—a set of computational methods addressing optimization problems as a continuous learning process—shows relevance for precision dosing with high flexibility for dosing rule adaptation and for coping with high dimensional efficacy and/or safety markers, constituting a relevant approach to take advantage of data from digital health technologies. RL can also support contributions to the successful development of digital health applications, recognized as key players of the future healthcare systems, in particular for reducing the burden of non-communicable diseases to society. RL is also pivotal in computational psychiatry—a way to characterize mental dysfunctions in terms of aberrant brain computations—and represents an innovative modeling approach forpsychiatric indications such as depression or substance abuse disorders for which digital therapeutics are foreseen as promising modalities.

## 1 Reinforcement learning for precision dosing

Precision dosing, or the ability to identify and deliver the right dose and schedule (i.e. the dose and schedule with highest likelihood of maximizing efficacy and minimizing toxicity), is critical for public health and society. Precision dosing is not only important for marketed drugs to reduce the consequences of imprecise dosing in terms of costs and adverse events; but also for therapeutics in development to reduce attrition, often related to the challenge of precisely characterizing the therapeutic window due to a suboptimal understanding of drug-response variability. Achieving the benefit to society of precision dosing requires the identification of the main drivers of response variability, as early as possible in the drug development process, and the deployment into clinical practice through an infrastructure designed for real-time dosing decisions in patients (Maxfield and Zineh, 2021; Peck, 2021).

Model-based approaches to clinical pharmacology, also known as clinical pharmacometrics (PMX) play a critical role in precision dosing. First, they contribute to the identification of the determinants of response variability through quantitative analysis of pharmacokinetic (PK) and pharmacodynamics (PD) relationships, and second, they constitute a central part of the infrastructure providing a simulation engine, predicting individual patient’s response to a dose, and from which optimal dosing is identified through reverse engineering. Often this reverse engineering comprises two steps: first the PMX model’s individual parameters are calculated through Bayesian inference, i.e. through the calculation of the mode of posterior distribution (maximum a posteriori or MAP); second, an optimal dosing scheduling is calculated, often *via* an heuristic approach through simulating various feasible dosing scenarios on inferred individuals model’s instances.

Many examples exist in literature describing relevant PKPD models for precision dosing. For instance, in oncology, a model describing the time course of neutrophils following chemotherapy treatment is an ideal candidate for optimizing chemotherapy delivery (see (Friberg et al., 2002) as an example). Studies have also reported clinical investigations of model-based precision dosing approaches. For instance, the clinical study “MODEL1” was a phase I/II trial and a clear clinical attempt of a personalized dosing regimen of docetaxel and epirubicin patients with metastatic breast cancer and was shown to lead to improved efficacy-toxicity balance (Henin et al., 2016).

Reinforcement learning (RL) was also used for precision dosing. Still in oncology, Maier et al. extended the classical framework of model-driven precision dosing with RL coupled or not with data assimilation techniques (Maier et al., 2021). Previously, RL applications—although without clinical confirmation—were developed for brain tumors (Yauney and Shah, 2018) based on a model of tumor size response to chemotherapy (Ribba et al., 2012). We have recently evaluated the performance of RL algorithms for precision dosing of propofol for general anesthesia and for which a meta-analysis showed that the monitoring of the bispectral index (BIS)—a PD endpoint—contributes to reduce the amount of propofol given and the incidence of adverse reactions (Wang et al., 2021). In (Ribba et al., 2022), we performed a theoretical analysis of propofol precision dosing confronting RL to hallmarks of clinical pharmacology problems during drug development, i.e. the low number of patients and tested dosing regimen, the incomplete understanding of the drivers of response and the presence of high variability in the data.

While RL does not present as a universal solution for all types of precision dosing problems, it is an interesting modeling paradigm worth exploring. In comparison to the way PMX traditionally addresses precision dosing, RL presents several advantages. First, the possibility to take into account high dimensional PKPD variables while classical model-based approaches are often limited to a low number of variables (plasma concentration and one endpoint). In doing so, it represents an opportunity for the integration of digital health data such as from wearable devices or digital health technologies in general. Second, the definition of the precision dosing policy in a dynamic and adaptable manner through the continuous learning of the algorithm through real and simulated experience (data). RL is an approach by which both the underlying model and the optimal dosing rules are learnt simultaneously while for classical approaches, these represent two sequential steps: in other words, the consequence of the dose does not influence the model structure. Recently, studies have been published illustrating methodologies for adapting PKPD model structures through data assimilation (Lu et al., 2021; Bram et al., 2022). While high dosing frequency is not a prerequisite condition for the applicability of RL to precision dosing, this approach is well suited when the solution space of dosing is large, making heuristic approaches to find optimal dosing solutions inadequate. In our example on propofol, dosing could happen every 5 s so over a short period of 2 min, the space of solutions to explore when considering dichotomous dosing even is greater than 16 million possibilities.

RL is at the crossroads between two scientific fields. First, the field of learning by trial and error that started with the study of the psychology of animal learning and second, the field of optimal control (Sutton and Barto, 2018). RL are often formally described with Markov Decision Process or MDP which includes all important features a learning agent should have, namely, being able to sense the environment, being able to take action and have clarity on the goal. In RL, a learning agent takes an action and, as a result, transitions from one state to another. After each action taken, the interaction between the agent and its environment produces a reward. The goal of the RL problem is to map actions to situations (state), i.e. knowing which actions to take in each state to maximize the accumulated reward. As long as the optimization problem can be formulated within the MDP framework, RL can be applied and its efficiency explored.

For precision dosing of propofol, the state can be represented by a table, an approach also called tabular solution methods. In the next two sections, the state will be defined by a continuous function. The reward was determined based on the value reached by the BIS as a direct consequence of the action taken: the closer the BIS to the target, the higher the reward. Finally, given the theoretical study, the true PKPD model (linking the dose application to BIS) was used as an experience (data) generator. The left column of Table 1 summarizes the characteristics of the application of RL to the propofol precision dosing problem.

**TABLE 1:** Main characteristics of RL algorithm implementation to the precision dosing of pharmacological interventions (left column); the precision dosing of digital intervention (middle column); and computational psychiatry (right column). While there are multiple similarities between the precision dosing of pharmacological and digital interventions, the application of RL in computational psychiatry shows as a paradigm shift. RL computational machinery is not deployed as a technical approach to address the optimal control problem of precision dosing but is fitted to (cognitive task) data assuming the algorithm itself presents mechanistic similarities with how brain’s participants functioned during the task.

	Precision dosing of a pharmacological intervention	Precision dosing of a digital intervention	Computation psychiatry
Study case [References]	Optimal dosing of propofol administration (Ribba et al., 2022)	Just-in-time-adaptive-intervention for HeartSteps, mobile app aimed at reducing physical inactivity (Liao et al., 2020)	Population analysis of signal-detection task in anhedonic subjects (Huys et al., 2013)
Type of RL solution	Tabular	Continuous	
State	*Is directly linked to the state of the patient*		*Is linked to the situation the participant to the task is presented with and based on which an action must taken*
	PK drivers and/or PD endpoint such as the BIS	Contextual drivers (e.g. weather conditions, time of the day) and patient-related status derived from wearable device equipment	Belief of the correctness (weight) of each stimuli present in the task
Action	Dose or not	Dose (walking suggestion message) or not	Participant’s answer choice
Reward	*Defined to enable the algorithm converging to the optimal dosing solution*		*Corresponds to whether the answer is correct or wrong*
	Simple function of BIS leading to high reward when actual BIS is close to its target	Step count in the 30 min window after each decision time	Automatically derived from the answer as per task design and setup
Use of simulated experience?	*Yes*		*No*
	The true underlying PKPD model is used	Linear model assimilating real data	No need for simulated experience, RL algorithm is mapped to the trial-by-trial data
Algorithm	Temporal difference Q-learning	Thomson Sampling	Temporal difference Q-learning
Free parameters	*Used to calibrate model of patient’s response to dosing event*		*Used to calibrate RL algorithm*
	Parameters of the PKPD model	Parameters of the linear model for reward prediction under alternative dosing scenarios	Learning rate and reward sensitivity parameter

The minimal set of RL characteristics makes it a very flexible paradigm, suitable for a large variety of problems. Herein, we will in fact illustrate this flexibility by illustrating how this framework can be viewed as a bridge between a priori distinct areas such as precision dosing of pharmacological drugs, digital health and computational psychiatry.

In the appendix, we propose to demystify how RL algorithms—such as temporal difference Q-learning, repeatedly mentioned here—work, taking a simple illustration from video gaming.

## 2 Reinforcement learning in digital health

For several years, many reports have indicated the key importance of digital health for reducing the burden to society of non-communicable diseases such as cardiovascular, diabetes, cancer or psychiatric diseases, in part due to the aging of the population and—paradoxically—the success of pharmacologically-based interventions in increasing life expectancy while being affected by pathological conditions (Fleisch et al., 2021). Prevention and interventions targeting lifestyle are essential tools to address this societal challenge of ever-growing importance as our healthcare systems risk collapse under cost pressure.

In 2008, it was estimated that physical inactivity causes 6% of the burden of coronary heart disease, 7% of type II diabetes, 10% of breast cancer and 10% of colon cancer and overall the cause of more than 5.3 million of the 57 million deaths which occurred that year (Lee et al., 2012). In that study, the authors also estimated that with 25% reduction of physical inactivity, 1.3 million of deaths could be averted every year. Given the constant increase of smartphone coverage worldwide, it is natural to think of mobile health technologies to support healthy lifestyle habits and prevention. The thinktank Metaforum from KU Leuven dedicated its position paper 17 on the use of wearables and mobile technologies for collecting information on individual behavior and physical status—combined with data from individual’s environment—to personalize recommendations (interventions) bringing the subject to adopt a healthier lifestyle (Claes, 2022).

When the intervention is intended to have a therapeutic benefit, it falls in the field of digital therapeutics when associated with demonstration of clinical effectiveness and approved by regulatory bodies (Sverdlov et al., 2018). This point of junction between digital health applications and pharmacological drugs represents a ground for attempting to reframe PMX—a recognized key player in the development of the latter—as a key support to the development of the former, in particular when it comes to precision dosing for digital health.

The precision dosing of digital therapeutics overlaps with the concept of just-in-time adaptive intervention or JITAI (Nahum-Shani et al., 2018). In the mobile technology literature, JITAI has been primarily considered as a critical topic for increasing adherence and retention of users; but within a therapeutic perspective, it should encompass both the topic of adherence and retention to the therapeutic modality and the topic of its optimal dosing in order to maximize clinical benefit. For clarity, these two different learning problems should be distinguished as many existing applications focus primarily on the first one. For example, a growing number of mobile applications developed under the concept of virtual coaching aim to optimize the design of the interventions (time and content, e.g. messages sent by the app to the users with the form of a prompt appearing on a locked screen) to incite the user to take actions. HeartSteps was designed to encourage user to increase their physical activity and where content delivery, such as tailored walking suggestion messages, is optimized with an RL algorithm (Liao et al., 2020). Here, RL is used to address the first learning problem: How to deliver the content so that the user is doing what is recommended. We each need different forms of prompting and potentially different forms of exercise to increase our physical activity. Overall, this problem is similar to that of adherence to a pharmacological regimen. But a second problem is: what is the right dose of the desired intervention? In other words: How many steps is optimal for each patient? This is the usual precision dosing problem for drugs and there is a clear opportunity for digital health applications to extend the domain of application of JITAIs to that problem as well.

One of the particularly interesting aspects of the research on RL algorithms for HeartSteps is that, beyond the innovative nature of the work purely related to the design of personalized interventions, it also includes ways to objectively evaluate its efficiency. An experimental design called micro-randomized trial (MRT) is proposed as a framework to evaluate the effectiveness of personalized *versus* non-personalized interventions (Klasnja et al., 2015; Qian et al., 2022). The principle of MRT is to randomize the interventions multiple times for each subject. Statistical approaches have been studied to leverage MRT-derived data in order to inform treatment effects and the response variability (Qian et al., 2020). In the theoretical propofol example described in the previous section, we used the true PKPD model to simulate experience. In the real-life RL application of HeartSteps, the authors had the objective to design a method for learning quickly and for accommodating noisy data (Liao et al., 2020). To address these points, the authors used a simulation engine to enhance data collected from real experience and this simulation engine was built with simple linear models. Precisely, the authors modeled the difference in reward function under alternative dosing options with low dimensional linear models, which features were selected based on retrospective analysis of previous HeartSteps data and based on experts’ guidance. The precision dosing problem was addressed using posterior sampling *via* Thompson-Sampling, identified as performant in balancing exploration and exploitation (Russo and Van Roy, 2014; Russo et al., 2018). The definition of the state was based on several individual’s features including contextual information or sensor data from wearable devices while the reward was defined as the step counts within 30 min after the “dosing” event. The middle column of Table 1 summarizes the main characteristic of RL application to this problem.

## 3 Reinforcement learning in computational psychiatry

Like mechanistic modelling, computational psychiatry refers to a systems approach aimed at integrating underlying pathophysiological processes. However, while mechanistic modelling efforts typically use multiscale biological processes as building blocks, some models that fall within the remit of computational psychiatry (such as RL) use different types of building blocks, and in particular brain cognitive processes.

Model-based approaches have shown relevance for addressing major challenges in neuroscience (see (Conrado et al., 2020) for an example for Alzheimer disease). Quantitative systems pharmacology and mechanistic-based multiscale modelling are, in particular, associated with major hopes while acknowledging significant challenges such as the lack of quantitative and validated biomarkers, the subjective nature of clinical endpoints and the high selectivity of drug candidates not reflecting the complex interactions of different brain circuits (Geerts et al., 2020; Bloomingdale et al., 2021). These challenges are equally valid for attempting to address psychiatric conditions. This can partly explain the efficiency of non-pharmacological interventions, such as targeted psychotherapy approaches, recognized as one of the most precise and powerful approaches (Insel and Cuthbert, 2015).

The efficiency of such interventions is a testimony of how the brain’s intrinsic plasticity can alter neural circuits. Some (discursive) disease models—with a focus on systems dimensions–propose new perspectives in the understanding of such conditions. For instance, it has been reported that emotion-cognition interactions gone awry can lead to anxiety and depression conditions; with anxious individuals displaying attentional-bias toward threatening stimuli and have difficulty disengaging from it (Crocker et al., 2013). Further data-driven understanding—at the systems level—is key to increase the likelihood of success of such non-pharmacological interventions, as it is equally the case for research and development of pharmaceutical compounds (Pao and Nagel, 2022). Such data-driven understanding can be integrated in the design of relevant non-pharmacological interventions, with some of them known to be amenable to digital delivery through, for instance, digital therapeutics (Jacobson et al., 2022).

A precision medicine initiative—precision psychiatry—has been initiated for psychiatric indications, such as major depression or substance abuse disorder, constituting a major part of non-communicable diseases (Insel and Cuthbert, 2015). The core idea of precision psychiatry lies in the reframing the diagnosis and care of affected subjects by moving away from a symptom-based to a data-driven categorization through a focus on system dimension *via* integration of data from cognitive, affective and social neuroscience, overall shifting the way to characterize these conditions in terms of brain circuits (dys-)functioning. This concept materialized in proposing the Research Domain Criteria (RDoc) in 2010 (Insel et al., 2010) as a framework for research in pathophysiology of psychiatric conditions.

Integrating into a multiscale modelling framework, data from cognitive, affective and social neuroscience is an objective of computational psychiatry, defined as a way to characterize mental dysfunction in terms of aberrant computation in the brain (Montague et al., 2012). Not surprisingly, by its mimicking of human and animal learning processes, RL plays a key role in computational psychiatry. RL in computational psychiatry proposes to map brain functioning in an algorithmic language offering then the possibility to explore, through simulations, the dysfunctioning of these processes as well as the theoretical benefit of interventional strategies. Two examples will be further developed here and the readers can refer to (Seriès, 2020) for an overview of more computational psychiatry methods, models and study cases.

In a RL framework, actions by the learner are chosen according to their value function, which holds the expected accumulated reward. The value function is updated through experience using feedback from the environment to the action taken. This update is also called temporal difference. An analogy has been drawn between this temporal difference and reward-error signals carried by dopamine in decision-making. Temporal difference reinforcement learning algorithms learn by estimating a value function based on temporal differences. The learning stops as this different converges to zero (see Supplementary Material for further details). Such a framework can be used to reframe addiction as a decision-making process gone awry. Based on the observation that addictive drugs produce a transient increase in dopamine through neuropharmacological mechanisms, the proposed model assumes that an addictive drug produces a positive temporal difference independent of the value function so that the action of taking drug will be always preferred over other actions (Redish, 2004). This model provides a tool to explore the efficiency of public health strategies. For instance, the model proposes some hypotheses to explain the incomplete success of strategies based on offering money as an alternate choice from drug intake.

RL models are used for the analysis of data of cognitive tasks and in particular tasks related to decision-making. Instead of focusing on the summary statistics of such tests (e.g, total number of errors), RL-based approaches allow for the integration of trial-by-trial data similarly to what model-based approaches typically do—with longitudinal data analysis—to better decipher response variability *via* the characterization of PK and PD processes. In the same way, trial-by-trial data can be leveraged to estimate RL-model based parameters which, in turn, can be compared to clinical endpoints such as measures of symptom severity to disentangle the role of brain circuit mechanisms overall contributing to a better understanding of response variability. RL for cognitive testing data in psychiatric populations is a complete paradigm change with respect to its application for precision dosing problems. While–in the two previous examples—RL was used to solve the problem of optimal dosing, now the RL algorithm is mapped to neuro-cognitive processes. Quantitatively characterizing these processes for each patient (estimating parameters from RL algorithms) is proposed as a methodology for extracting relevant information towards disease characterization and thus, response variability.

In (Huys et al., 2013), the authors use RL models to analyse population data of a behavioural test (signal-detection task) to study aspects of anhedonia—a core symptom of depression—related to reward learning. The authors proposed a RL model based on Q-learning update integrating two parameters: the classical learning rate and a parameter related to reward sensitivity modulating the percentage of the reward value actually contributes to the update of the Q value function. By performing a correlation analysis of the inferred parameters with anhedonic depression questionnaire, the authors found a negative correlation between the reward sensitivity but no correlation with the learning rate. Overall, these results led to the conclusion that the sensitivity to the reward and not the learning rate could be the main driver explaining why in anhedonic individuals, reward has less impact than in non-anhedonic individuals. Unravelling these two mechanisms is important for the planning of successful digital, behavioural and pharmacological strategies. The right column in Table 1 depicts the summary characteristics of RL applied to that study.

## 4 Conclusion

In this perspective, we have illustrated the flexibility of RL framework throughout the described applications in precision dosing, digital health and computational psychiatry and with that have demonstrated the benefit for the modeling community to become familiar with these approaches. The contrary is also true, and the field of precision digital therapeutics and computational psychiatry can benefit much from a proximity to the PMX community.

First, PMX methods could make RL even better. The field of computational psychiatry could benefit from input from the PMX community when it comes to statistical aspects related to parameters inference and clinical endpoint modelling. Two areas for which PMX has adopted as its state-of-the-art, population approach (with powerful algorithms such as stochastic approximation expectation-maximization algorithm (Lavielle, 2014)) and joint modelling respectively.

**FIGURE 1:**
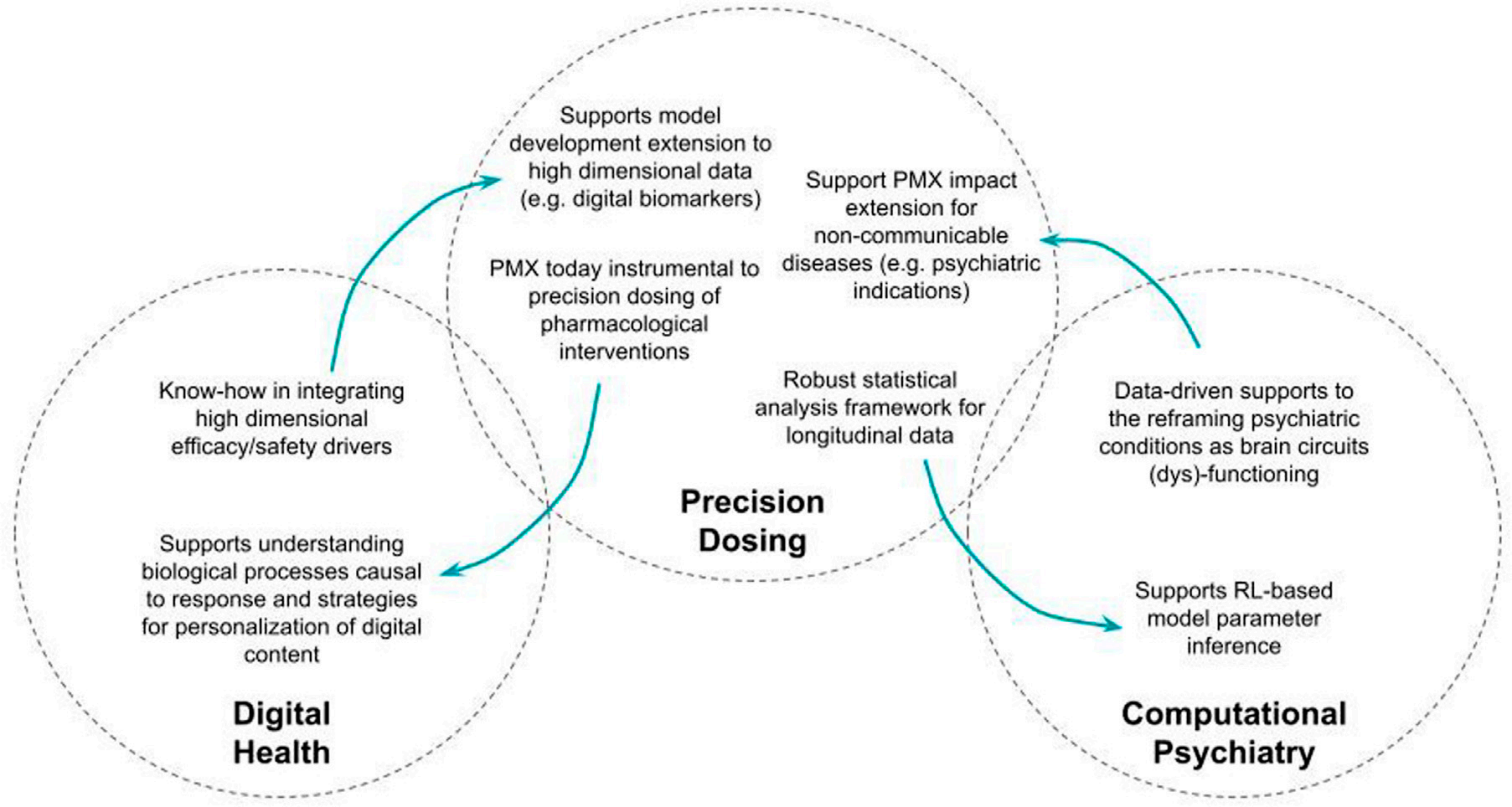
Illustration of the mutual benefits of increased permeability between model-based approaches to precision dosing and digital health, on one hand, and computational psychiatry on the other hand.

Second, the field of digital health should benefit from what constitutes one of the essential objectives of model-based drug development approaches, namely: elucidating response variability. It is particularly important for the successful development of digital therapeutic interventions to know how to characterize the efficacy and safety profiles and to know how to develop personalization strategies based on this understanding. The fact that it is about digital interventions should not prevent developers from prioritizing research in understanding underlying causal biological and (patho)-physiological processes of response, which will always be a key factor of successful therapy development, either pharmacological or not. Figure 1 proposes an illustration of these mutual benefits.

## 5 Legend

Table 1 **:** Main characteristics of RL algorithm implementation to the precision dosing of pharmacological interventions (left column); the precision dosing of digital intervention (middle column); and computational psychiatry (right column). While there are multiple similarities between the precision dosing of pharmacological and digital interventions, the application of RL in computational psychiatry shows as a paradigm shift. RL computational machinery is not deployed as a technical approach to address the optimal control problem of precision dosing but is fitted to (cognitive task) data assuming the algorithm itself present mechanistic similarities with how brain’s participants functioned during the task.

## Data Availability

The original contributions presented in the study are included in the article/Supplementary Material, further inquiries can be directed to the corresponding author.
