# Supplementary material for: Reinforcement learning as an innovative model-based approach: Examples from precision dosing, digital health and computational psychiatry
Source: Front Pharmacol. 2023 Feb 17;13:1094281. doi: 10.3389/fphar.2022.1094281 (PMC9981647; doi:10.3389/fphar.2022.1094281)
Supplement: Supplementary file 1 [file DataSheet1.docx]

Supplementary Material: How RL Algorithm Work

# Supplementary Data

In this appendix, we propose to explain simply the way reinforcement learning (RL) algorithm – such as temporal difference RL –work through playing a simple game: “tic-tac-toe”. The reader can refer to more complete explanations here [1].

Supplementary figure 1 shows an illustration of a situation while playing tic-tac-toe. To map the elements of the figure to a RL problem, we will define the state of the learning agent as the board available to the agent before being able to play. The action corresponds to placing an X in one of the available positions (for example playing on 9 and then playing on 1 to win the game). The reward is the consequence of the action taken. After playing on 9, the reward is null because the game continues but after playing 1 in the next state, the reward is positive (+1) as player X won the game.

The aim of the computer method will be to identify an optimal policy, which is knowing which action *a* to take (where to play) from any state *s* in order to maximize long-term return which is, in this game, to win or not loose.

We will define a function called $Q(s,a)$, the action-value function which holds the expected return taking action $a$ when in state $s$. The return being defined as the sum of all future rewards. If $Q$ is known, the solution is obvious in the sense that we just need to select the action $a$ where $Q(s,a)$ has the highest value: this is call the optimal policy.

The function $Q$ is estimated through experience. $Q$ learning with temporal different consists of estimating iteratively the $Q$ function by updating its value by new information (called temporal difference or TD) learned through a real experience or a model. The temporal difference being the difference between the new estimation of the return following the new experience and the old estimation:

$$new\_Q\left( s,a \right)=old\_\_Q\left( s,a \right)+TD$$

If the dataset contains the experience as depicted in Supplementary Figure 1, when playing on 9 and then playing on 1, the temporal difference will be the difference between the best expected return: so 0 (because there is no reward after playing 9) + 1 (because player X win after playing 1) and the previous estimation of $Q$:

$$new\_Q\left( s_{i},9 \right)=old\_\_Q\left( s_{i},9 \right)+\alpha\left[ 0+1- old\_\_Q\left( s_{i},9 \right) \right]$$

The information that from state $s_{i}$ there is a chance to win the game (reward +1) with future moves is back-propagated to that state. By training the algorithm with a large amount of trials, the $Q$ function can be estimated.

# Supplementary Figures and Tables

## Supplementary Figures


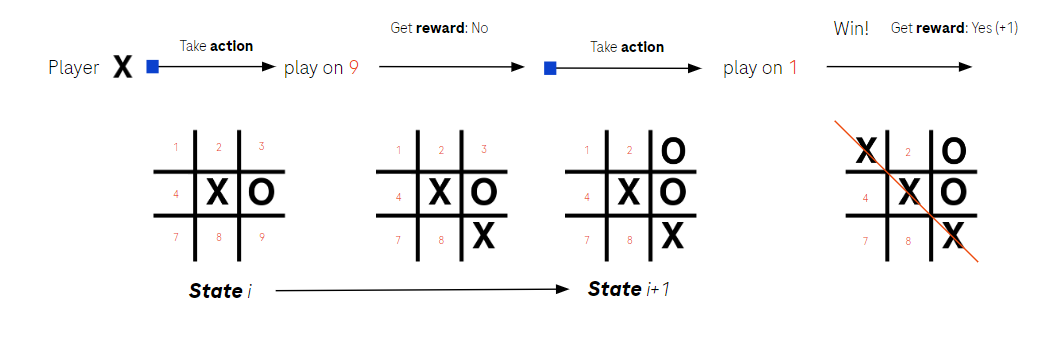


**Supplementary Figure 1.** Succession of actions, states and rewards in playing “tic-tac-toe”.

**References:**

1. Sutton, R. and A. Barto, *Reinforcement Learning: An Introduction*. Second edition ed. 2018.
